# Supplementary material for: Trends and characteristics during 17 years of naloxone distribution and administration through an overdose prevention program in Pittsburgh, Pennsylvania
Source: PLoS One. 2025 Oct 24;20(10):e0315026. doi: 10.1371/journal.pone.0315026 (PMC12551907; doi:10.1371/journal.pone.0315026)
Supplement: S1 File — (DOCX) [file pone.0315026.s001.docx]

SUPPLEMENTAL INFORMATION

**Trends and Characteristics during 17 Years of Naloxone Distribution and Administration through an Overdose Prevention Program in Pittsburgh, Pennsylvania**

*Submitted to: PLoS One*

Nabarun Dasgupta^1,3*^, Alice Bell^2^, Malcolm Visnich^2^, Maya Doe-Simkins^3^, Eliza Wheeler^3^, Adams L. Sibley^1^, Maryalice Nocera^1^, Amy E. Seitz^4^, Dorothy Chang^4^, Summer Barlow^4^, Zachary Dezman^4^, Jana McAninch^4^

1. University of North Carolina, Chapel Hill, North Carolina, USA

2. Prevention Point Pittsburgh, Pittsburgh, Pennsylvania, USA

3. Remedy Alliance For The People, Berkeley, California, USA

4. US Food and Drug Administration, Silver Spring, Maryland, USA

**INTRODUCTION**

**Table S1.** Current- and Previously-Approved Products Containing Naloxone Hydrochloride

| **Product Name** | **Year of Approval** | **Route of Administration** | **Other Comments** |
| --- | --- | --- | --- |
| **Approved treatments containing naloxone** | | | |
| NARCAN (NDA 16636, many generics available) | 1971 | Injection for IV, IM, SC. Available concentrations: 0.02 mg/mL, 0.4 mg/mL, and 1 mg/mL | Approved for use in entire pediatric range. |
| NARCAN | 2015 | 4 mg nasal spray | Nonprescription as of 3/2023 |
| KLOXXADO | 2021 | 8 mg nasal spray | Prescription |
| ZIMHI | 2021 | 5 mg prefilled syringe IM/SC | Prescription |
| RiVIVE | 2023 | 3 mg nasal spray | Nonprescription as of 7/2023 |
| REZENOPY | 2024 | 10 mg nasal spray | Prescription |
| **Approved but Currently Not Marketed** | | | |
| EVZIO | 2014 | 0.4 mg autoinjector IM/SC | Discontinued |
| EVZIO | 2016 | 2 mg autoinjector IM/ SC | Discontinued |
| NARCAN | 2017 | 2 mg nasal spray | Never marketed |

**METHODS**

**Data Preparation**

Initial data unpacking has revealed the presence of data entry keystroke errors. Because the people who entered the data are part of the research team, and because they have access to underlying paper records, we were able to remedy keystroke errors for a more complete dataset. Three kinds of data entry errors were identified: dates, categorical, and typographic. Missingness was also addressed. For maximum transparency, rectification of keystroke errors were done only through code-based replacement commands so that every edit can be tracked and replicated.

*Dates*

For example, a date that is listed as “February 30” was corrected to February 28 based on leap year and days of site operation. Date keystroke errors were identified with logic checks (e.g., date of naloxone administration after date of naloxone receipt), and reviewed with the PPPGH team to make adjudications.

*Categorical*

Categorical errors include double entry of values within a column and incorrect placement of data between columns. For on whom naloxone was used, but “self” and “you” were evident in the dataset and combined. These errors were evident with logic checks and corrected, with assistance from PPPGH staff as needed.

*Typographical Conventions*

Typological inconsistencies were corrected using code-based in-data replacement. Examples include using semi-colon (;) instead of comma (,) for categorical entry. Capitalization (“im” instead of IM) were also made consistent.

*Missing Data Handling*

The dataset showed low levels of missingness, less than 3% for most variables. Missingness was reported in Table 1, with complete case analysis in the case of missingness. The only imputation was if naloxone use date was missing because of the reliance in time series, in which case the median days between use and report (overall median 18 days) was imputed within each time block as a replacement.

Response behavior and adverse event analysis was limited to cases with known date of naloxone use ensure consistency with time series analyses.

*ORE per 100 Doses Dispensed*

*ORE per 100 doses dispensed* (as presented in main manuscript Figure 2) is a novel surveillance metric developed in the course of this research for the purpose of localized outbreak surveillance. Initially we tried to construct a analogous definition that would match the standard syndromic surveillance definition of *opioid-related ED-visits per 10,000 population* *per month*. Since the harm reduction organization did not have a definable geographic catchment area, we attempted using number of participants (new or returning); for the sake of simplicity we present new participants as the denominator in Figure 2.

However, we found that ORE per 100 doses dispensed was a more responsive metric, and yielded a time series curve that bore striking resemblance to local hospital ED data, as can be seen in the screen capture from the local opioid dashboard below. See Figure 2 in main manuscript to compare ORE per 100 Doses Dispensed by Prevention Point Pittsburgh to hospital emergency department visit volume in Allegheny County, Pennsylvania presented below.

Source: [**https://public.tableau.com/app/profile/pennsylvania.pdmp/viz/PennsylvaniaODSMPDrugOverdoseSurveillanceInteractiveDataReport/Contents**](https://public.tableau.com/app/profile/pennsylvania.pdmp/viz/PennsylvaniaODSMPDrugOverdoseSurveillanceInteractiveDataReport/Contents) (Accessed September 24, 2024).


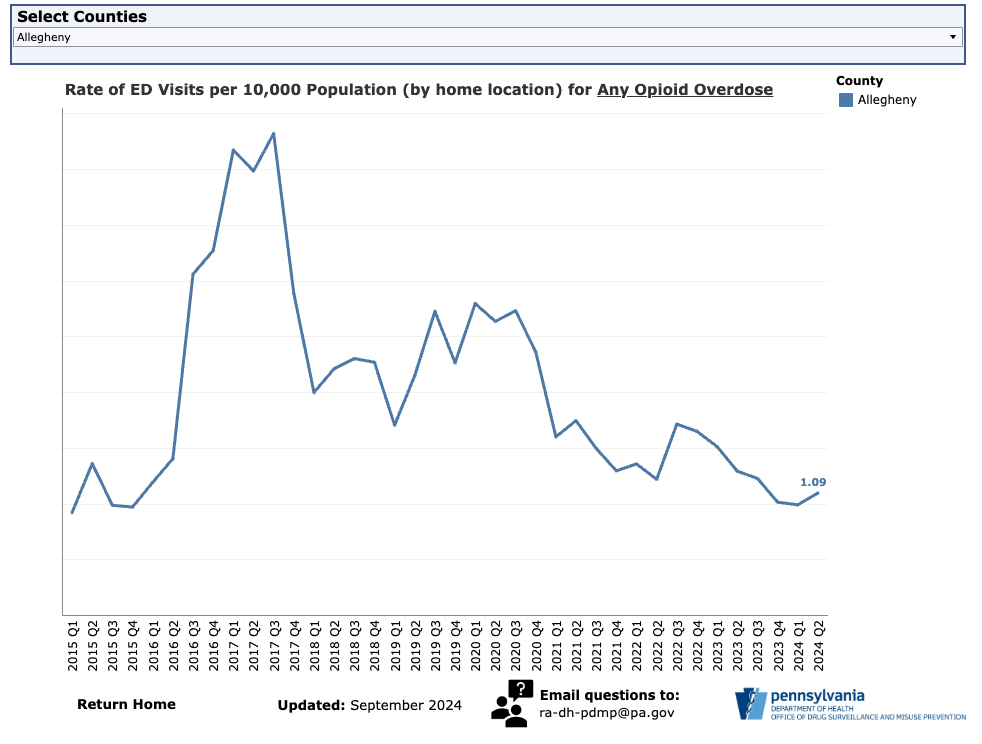


**RESULTS**

**Figure S1. Impact of legislation on new participant volume**

Monthly volume of new participants enrolled at Prevention Point Pittsburgh, from July 24, 2005 to January 24, 2023. New participant intakes increased sharply starting January 2015, after passage of state legislation enabling standing orders for naloxone distribution without the need to document trainings as clinical encounters. Using a linear spline with a knot at January 2015, regression models estimated an additional 56.2 (95% CI: 46.5, 65.8; Wald X^2^ 510, 3 df, p<0.001) new participants per month on average were enrolled at Prevention Point Pittsburgh after the law changed, jumping from 10 new participants per month before to 66 after. New participant intakes jumped from 10.4 per month (95% CI: 9.4, 11.4) before 2015, to 65.9 per month (95% CI: 60.7, 71.1) after the law was enacted (t-test 20.8, 103 Satterthwaite df, p<0.001).


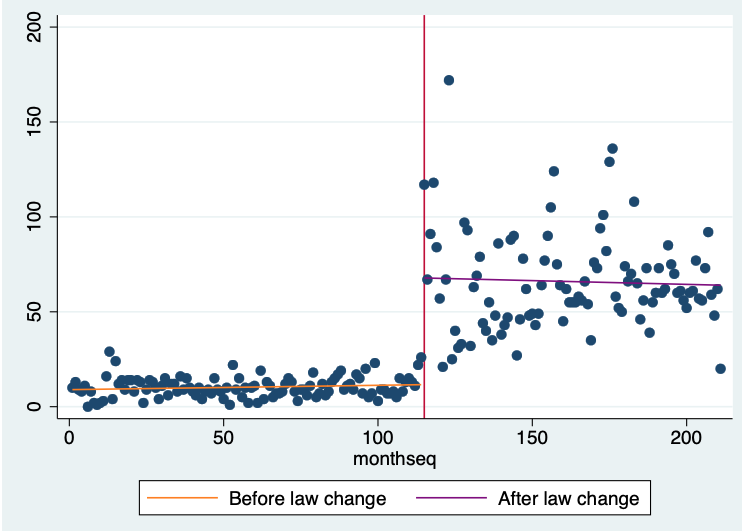


**Figure S2. Histogram of age at intake of new participants**

Age at intake of new participants enrolled at Prevention Point Pittsburgh, from July 24, 2005 to January 24, 2023.


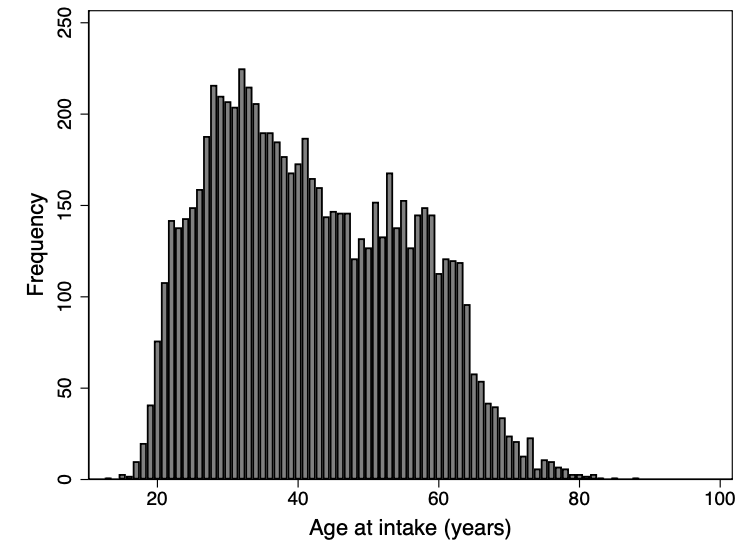


**Figure S3. Segmented regression for average age by month**


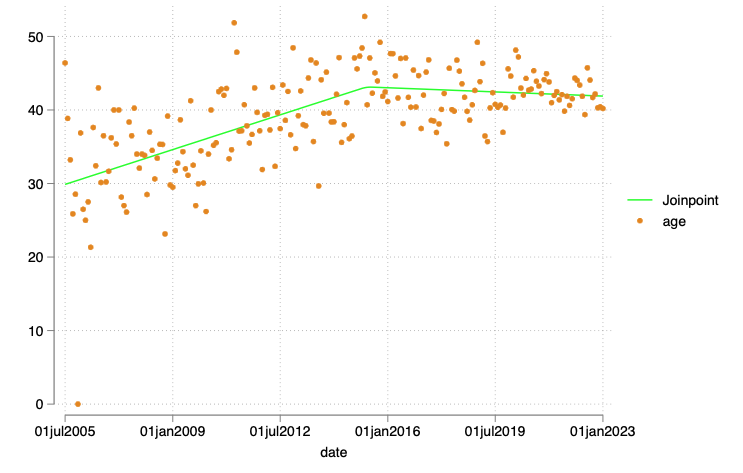


**Figure S4. Segmented regression for non-White new participants**


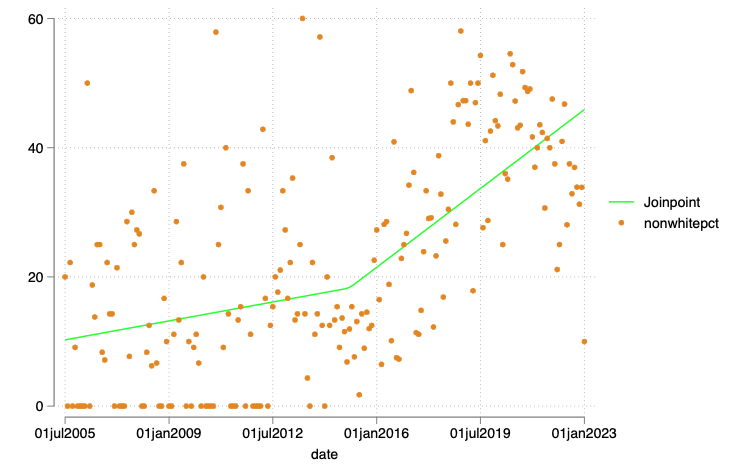


**Figure S5. Three-knot spline model for percent Black new participants**


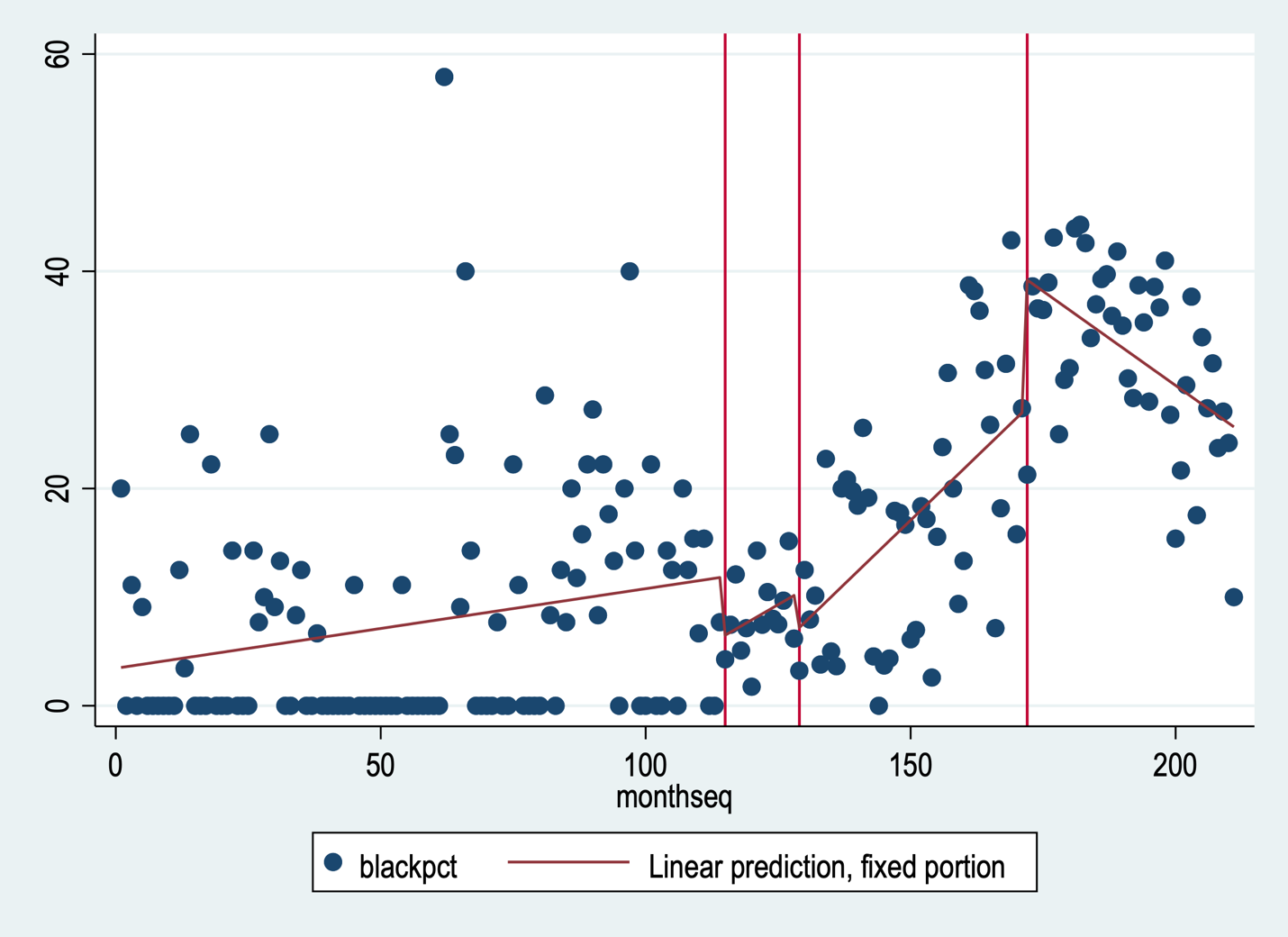


**Figure S6. Segmented regression analysis of “used it” as refill reason**


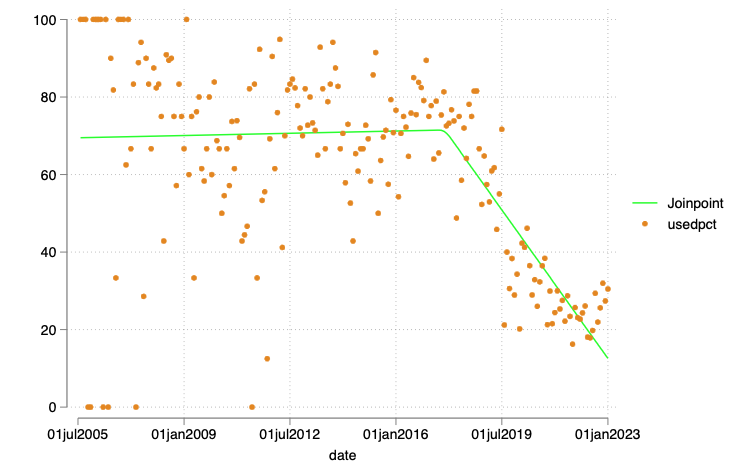


**Figure S7. Segmented regression on “gave away” as refill reason**


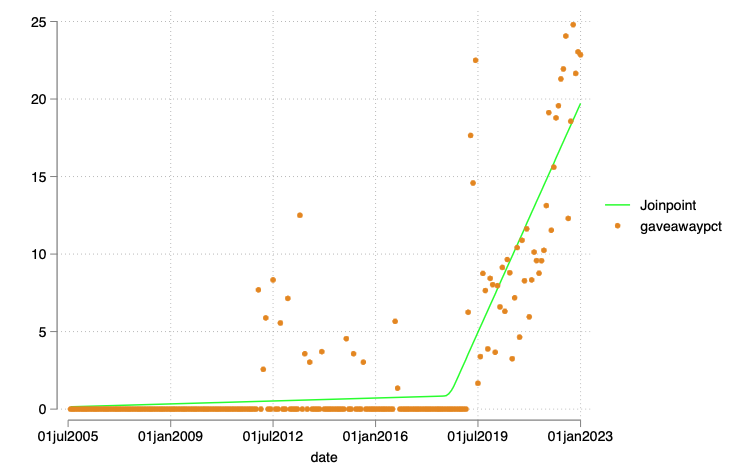


**Figure S8. Segmented regression analysis of “need extra kit” reason for refill**


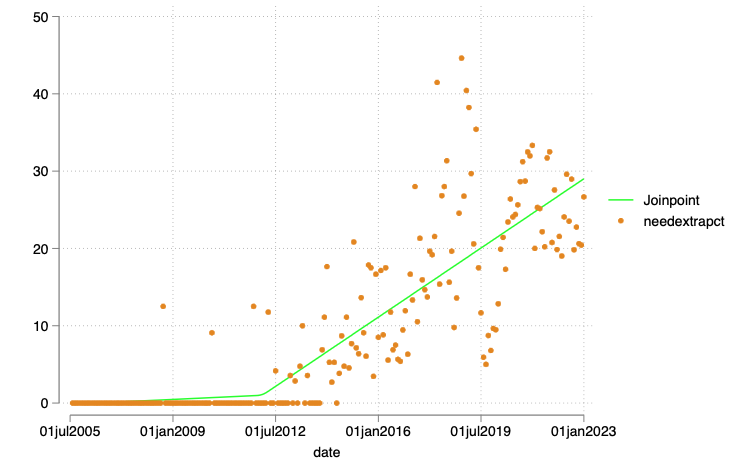


**Figure S9. Segmented regression analysis of naloxone used on “you” (reporter)**


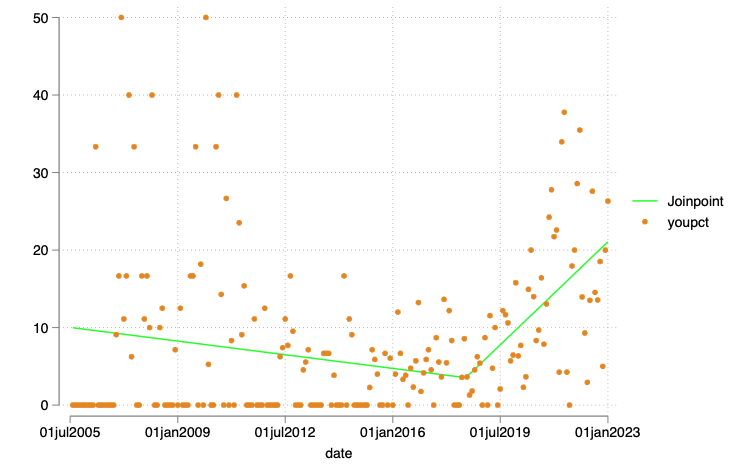


**Figure S10. Segmented regression analysis of naloxone administered to friends and acquaintances**


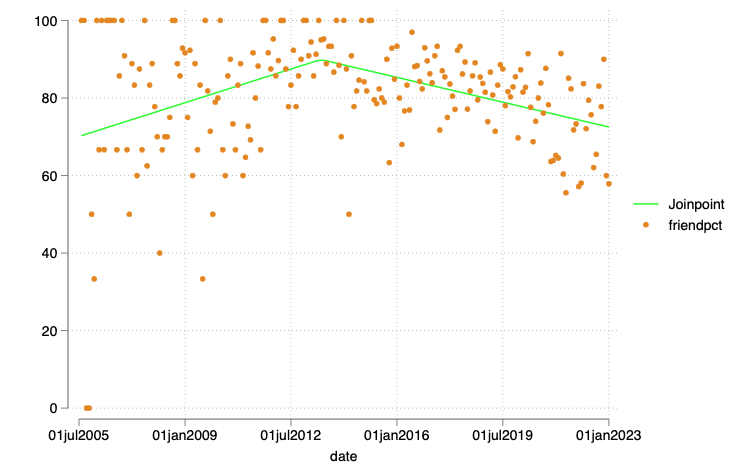


**Figure S11. Segmented regression analysis of naloxone administered to family**


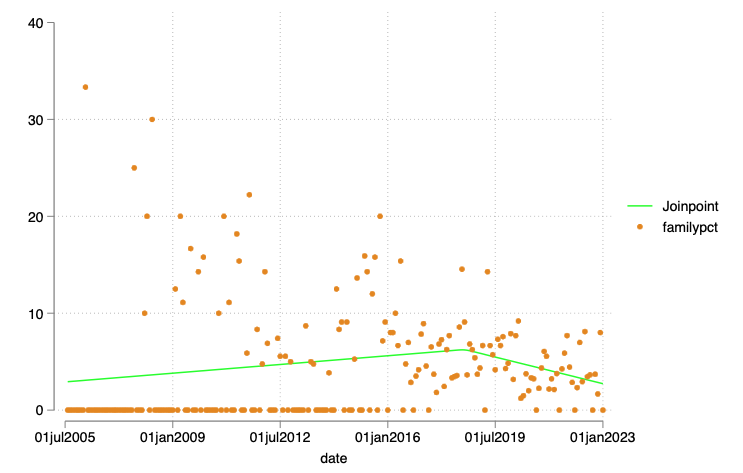


**Figure S12. Scatterplot overtime of naloxone administered to strangers**

**
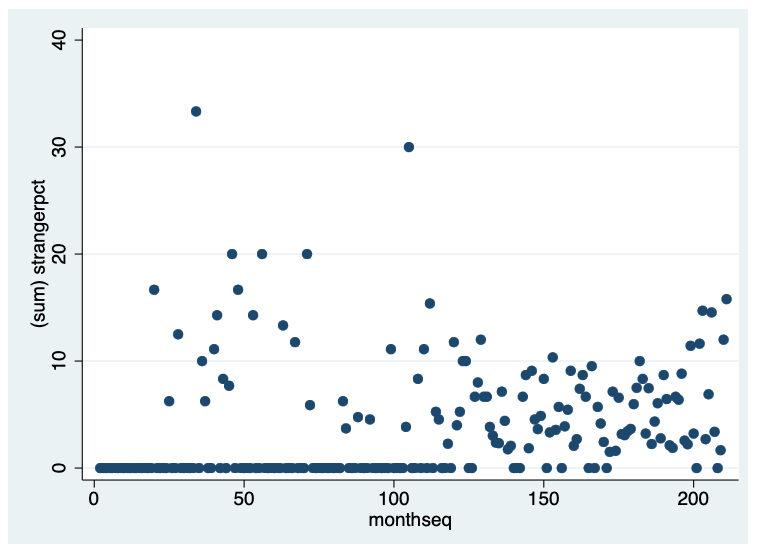
**

**Figure S13. Average number of naloxone doses per overdose response event, by month, all formulations**

**
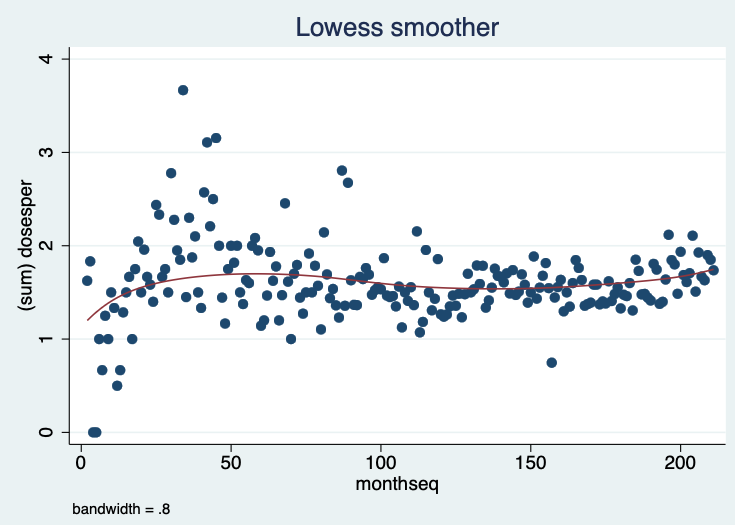
**

**Figure S14. Segmented regression analysis of rescue breathing performed during overdose response events**

**
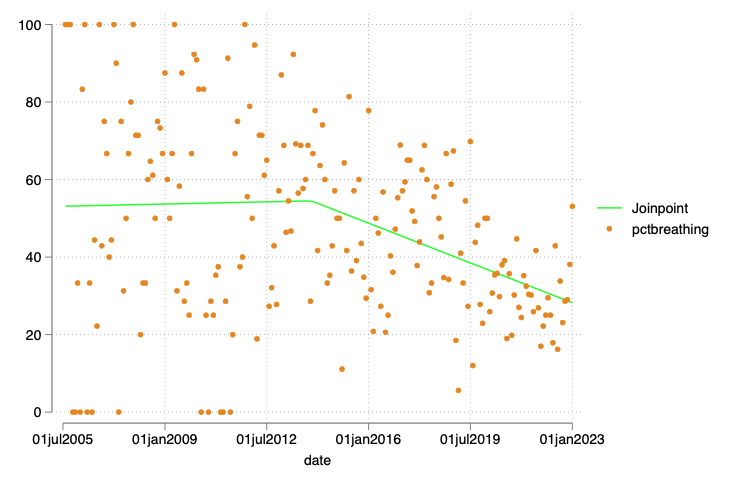
**

**Figure S15. Segmented regression analysis of chest compressions performed uring overdose response events**


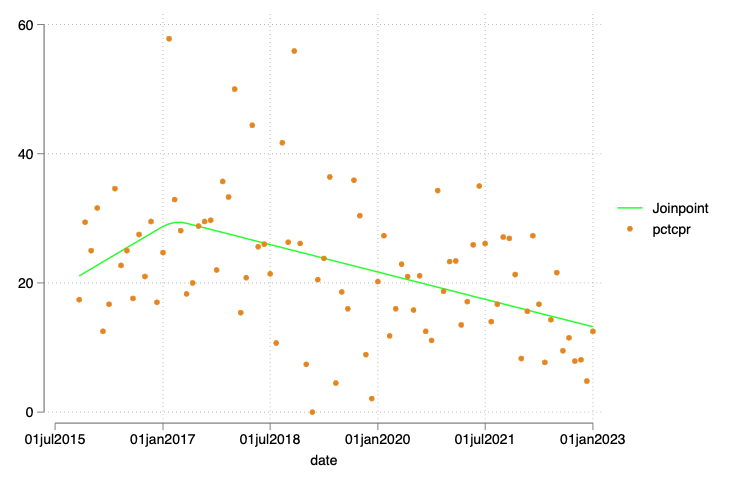


**Figure S16. Segmented regression analysis of 911 emergency services being called during overdose response events**

**
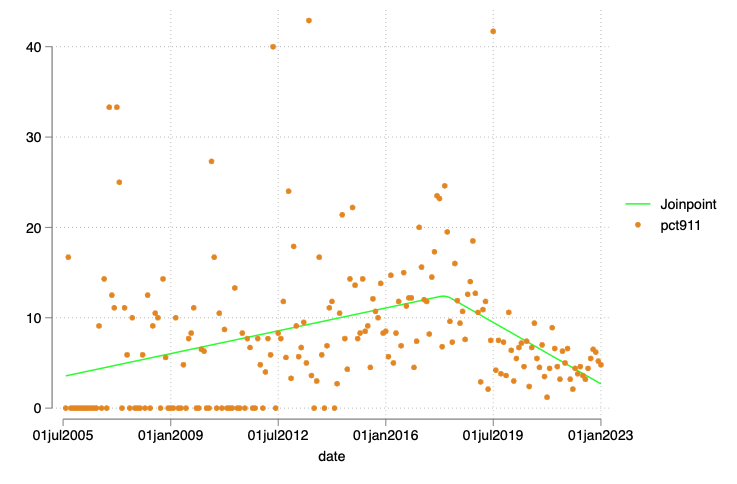
**

**Figure S17. Spline segmented regression analysis of 911 emergency services being called during overdose response events, before and after law change**

**
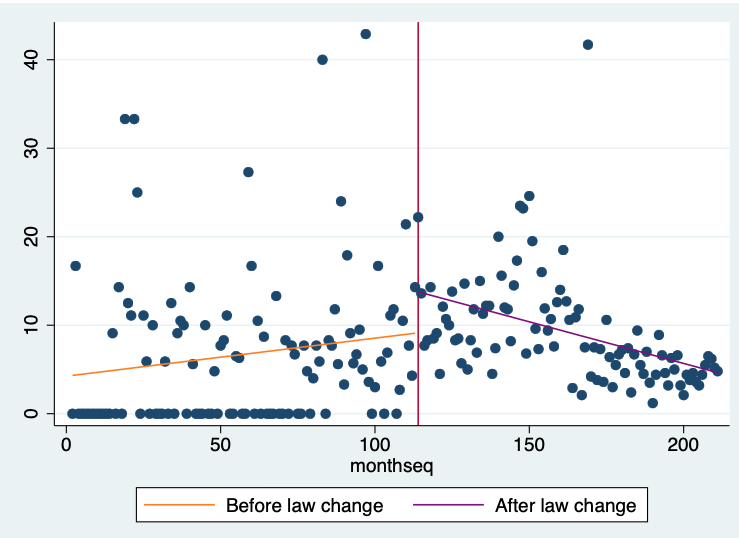
**

**Figure S18. Scatterplot of hospital transport during overdose response events**

**
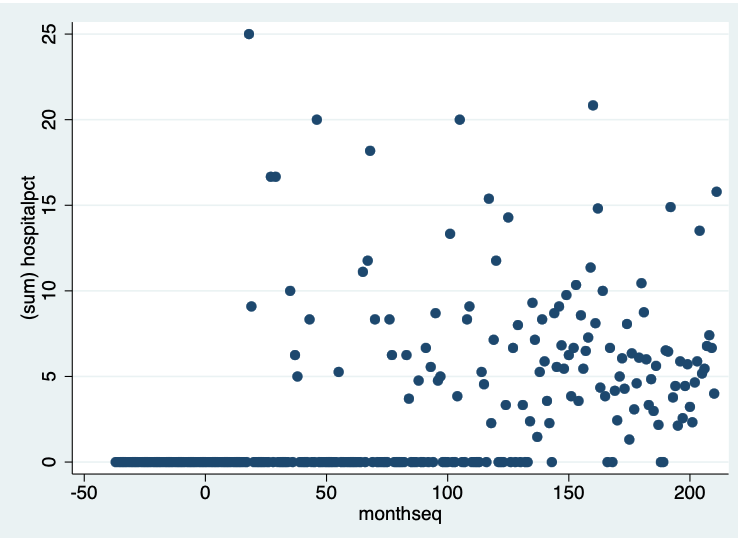
**

**Table S2. Key events impacting naloxone distribution and local drug market**

| **Date** | **Event** | **Implications** |
| --- | --- | --- |
| July 2005 | Naloxone distribution begins at Prevention Point Pittsburgh | New intervention added to syringe services |
| 2005 to 2015 | Local overdose deaths involve opioid analgesics and heroin[45,79] | Drug seizure data of stamp bags containing fentanyl or a fentanyl analogs was 2.1% in 2014 and rose to 17.1% in 2016. |
| October 2012 | Creation of the Naloxone Buyers Club | Access to low-cost naloxone via special arrangement with manufacturer; start of 1 mL vial distribution |
| November 2014 | Pennsylvania Act 139 | Allows standing order and third-party prescribing of naloxone; provides Good Samaritan protections for calling 911 |
| December 2014 | First training and naloxone distribution under Act 139 | Large group trainings for naloxone by Prevention Point Pittsburgh in surrounding communities |
| January 2015 | Mobile services started | Outreach to Black community, including hiring of staff from those areas. |
| 2016 | Naloxone provision to people leaving Allegheny County Jail | Increased naloxone carriage by population at risk for overdose due to recent incarceration |
| August 2016 | Naloxone nasal spray available | Begin purchasing by Allegheny County Department of Human Services |
| April 2020 | Start of COVID-19 pandemic | Sites remained open throughout the pandemic with certain accommodations (e.g., social distancing, masks, hand sanitizer) and new services (e.g., meals, COVID tests, vaccines). |
| May 2021 to August 2022 | Shortage of affordable 1 mL vials of naloxone | Due to manufacturing issues, low-cost naloxone supply was disrupted nationwide. State health department responded by providing naloxone nasal spray. |
| 2022 | Mobile buprenorphine clinic | Initially serving younger, White community. Brings in new participants for receiving naloxone also. |
| 2022 | Xylazine emerges in local drug supply | Sedating effects of xylazine believed to increase number of doses of naloxone administered due to continued unresponsiveness, despite restoration of respiration. Program staff messaging modified to emphasize rescue breathing and monitoring of respiratory rate prior to subsequent naloxone administration. |
| Fall 2023 | Over the counter naloxone becomes available after approval by FDA over the summer | Beyond the study’s observation period, but has potential to introduce additional sources of naloxone for community distribution. |

**Table S3. Adverse event frequency with naloxone dose titration**

With integer dosing as the referent group, IRRs below 1.0 represent the incidence rate ratio showing lower AEs with titration.

**Emesis**

|  | No Emesis | Emesis | Total | Rate  (per 100 reversals) |
| --- | --- | --- | --- | --- |
| Titration | 114 | 3 | 117 | 2.6 |
| Integer dosing | 2661 | 292 | 2953 | 9.9 |
|  | 2775 | 295 |  |  |

**IRR 0.26** (95% CI: 0.084, 0.80), Pearson X^2^ 6.9, p_exact_=0.006

**Angry**

|  | Not Anger | Anger | Total | Rate  (per 100 reversals) |
| --- | --- | --- | --- | --- |
| Titration | 116 | 1 | 117 | 0.85 |
| Integer dosing | 2642 | 311 | 2953 | 10.5 |
|  | 2758 | 312 |  |  |

**IRR 0.081** (95% CI: 0.011, 0.57) Pearson X^2^ 11.5, p_exact_<0.001

**Felt Sick**

|  | Not Felt Sick | Felt Sick | Total | Rate  (per 100 reversals) |
| --- | --- | --- | --- | --- |
| Titration | 112 | 5 | 117 | 4.3 |
| Integer dosing | 2668 | 285 | 2953 | 9.6 |
| Total | 2780 | 290 |  |  |

**IRR 0.44** (95% CI: 0.186, 1.051) Wald X^2^ 3.8, p=0.051

**Death**

|  | Not Death | Death |
| --- | --- | --- |
| Titration | 110 | 0 |
| Integer dosing | 2918 | 24 |

IRR = not derivable by model due to zero cell

**Shaking**

|  | Not Shaking | Shaking |
| --- | --- | --- |
| Titration | 117 | 0 |
| Integer dosing | 2926 | 27 |

IRR = not derivable by model due to zero cell
